# Supplementary figures and images for: Pangenome graph analysis reveals extensive effector copy-number variation in spinach downy mildew
Source: PLoS Genet. 2024 Oct 25;20(10):e1011452. doi: 10.1371/journal.pgen.1011452 (PMC11540230; doi:10.1371/journal.pgen.1011452)

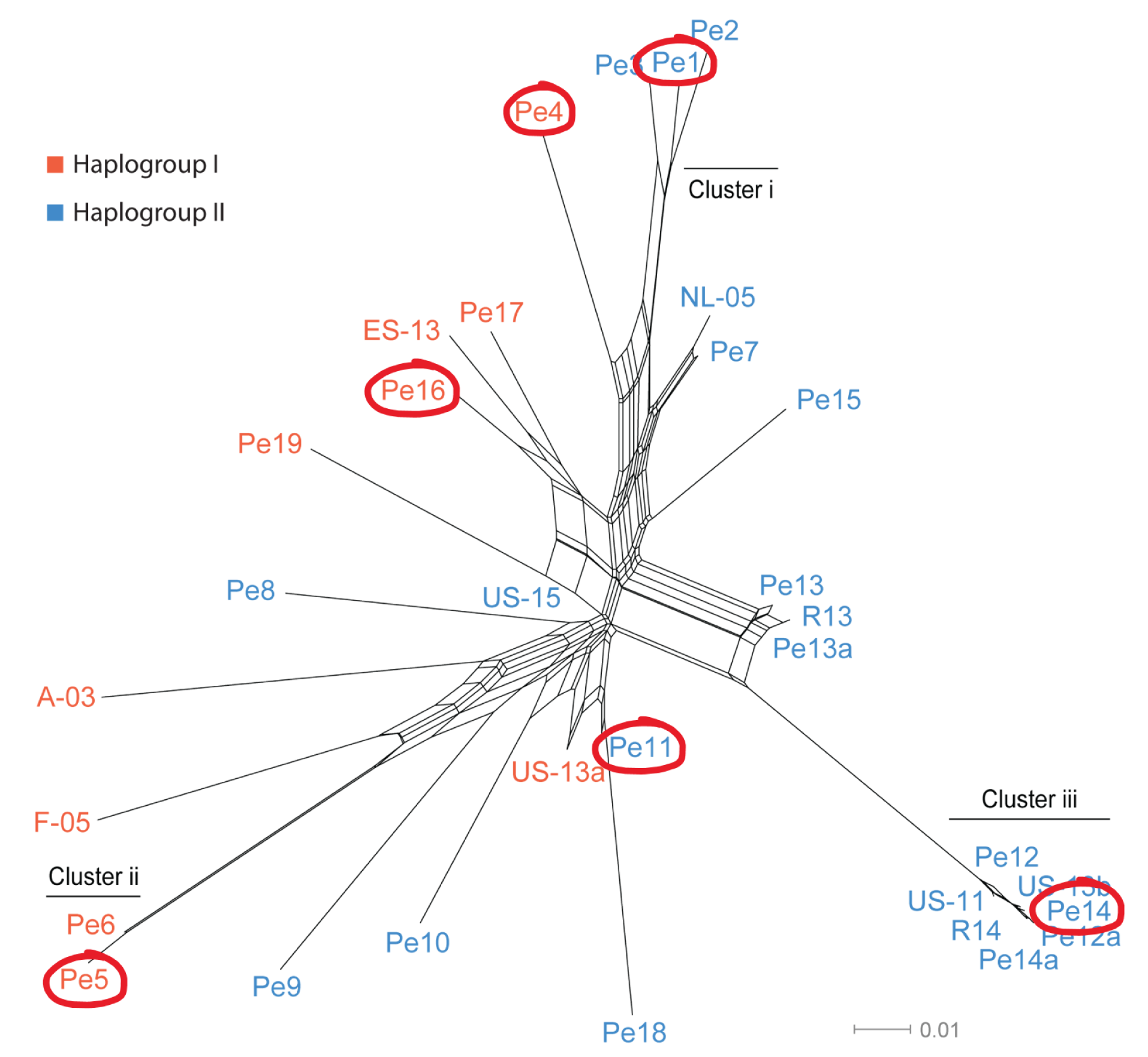

Supplement: S1 Fig — Neighbor-net phylogenetic network of P. effusa isolates that cover all known isolates based on a distance matrix of genome-wide nucleotide differences, containing 260,616 biallelic sites. The branch lengths are proportional to the calculated number of substitutions per site. The parallel edges connecting different isolates indicate conflicting phylogenetic signals. The six selected isolates are from six distinct races and are indicated with red circles. (TIF) [file pgen.1011452.s001.tif]

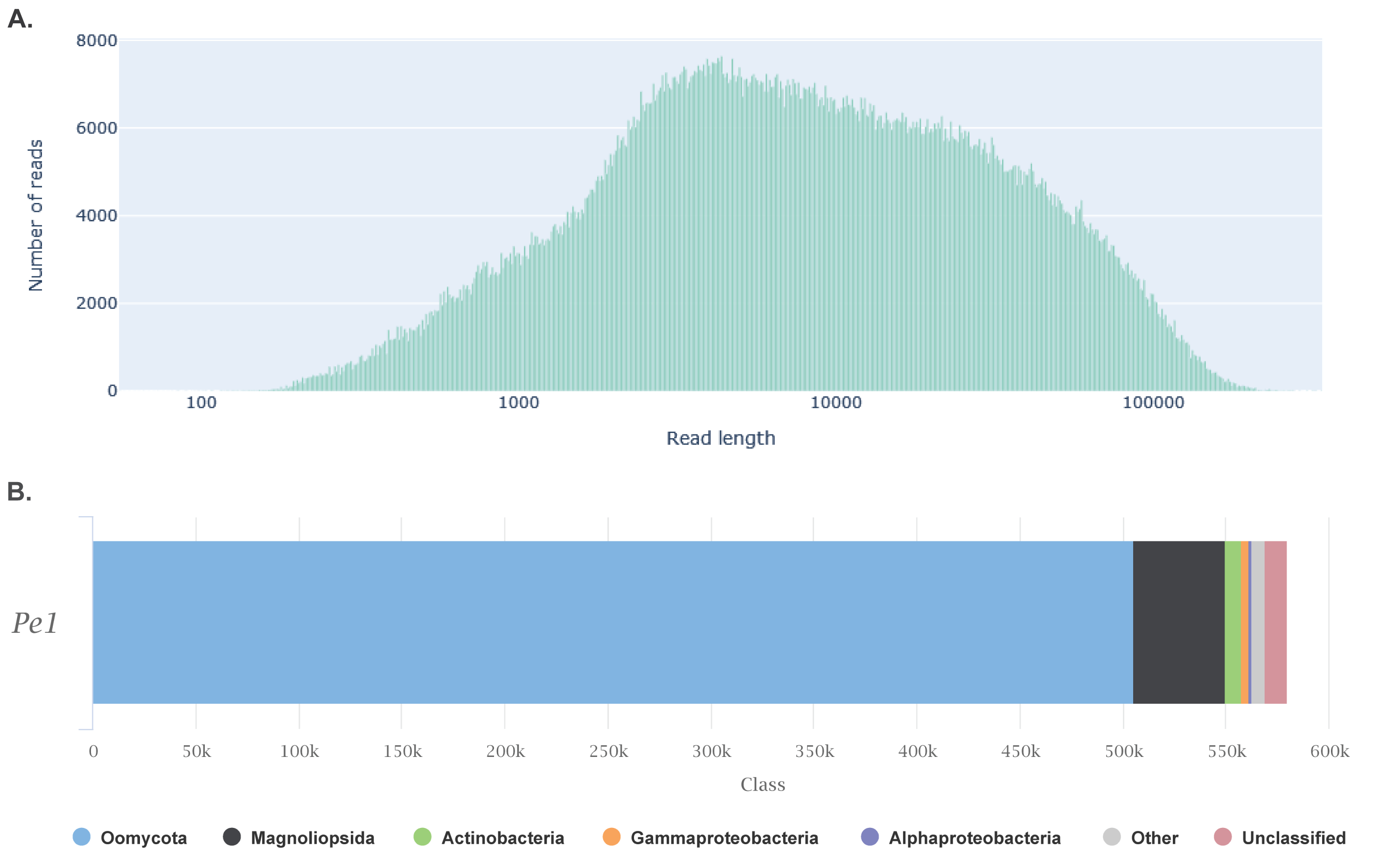

Supplement: S2 Fig — A. Weighted histogram of read lengths after log transformation. B. Phylogenetic classification of reads based on Kraken2 (Wood et al., 2019) and plotted with MultiQC (Ewels et al., 2016). All the reads classified as oomycote belong to P. effusa. (TIF) [file pgen.1011452.s002.tif]

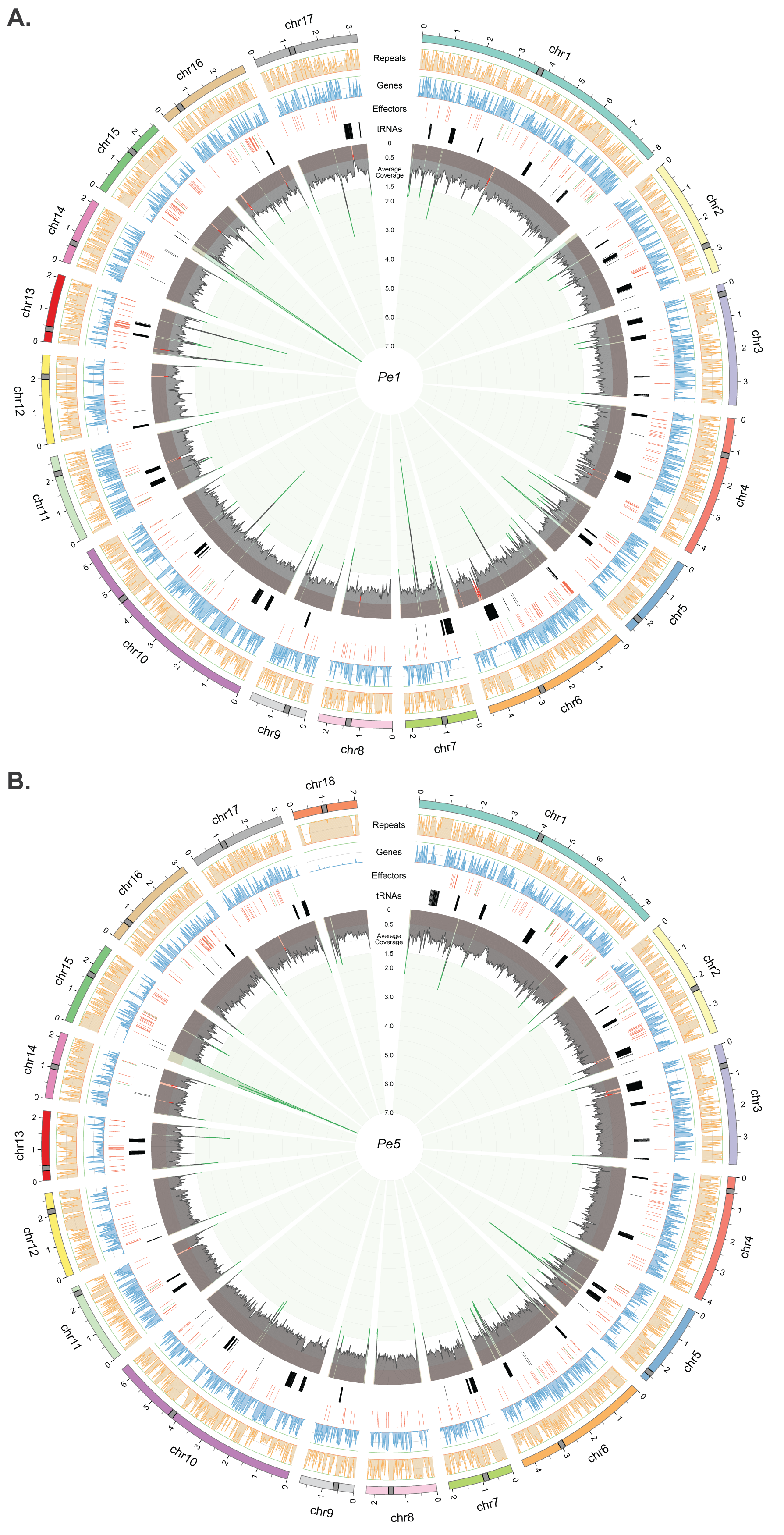

Supplement: S3 Fig — Individual tracks, starting from the outside to the inside: i) 17/18 chromosomes are shown in different colours, a grey rectangle point the location of the centromeres. ii) Line graph shows the coverage of repeat (orange) and gene (blue) content summarized in non-overlapping 20 kb windows. iii) Lines indicate the position of RXLR (red), CRN (green), and tRNA (black) genes. iv) Inverted line graph shows the nanopore average coverage of nanopore reads to the genome assembly. Coverage of 1 indicates a diploid coverage, 0.5 haploid coverage, and coverage higher than one indicates an underrepresentation of a repetitive region. (TIF) [file pgen.1011452.s003.tif]

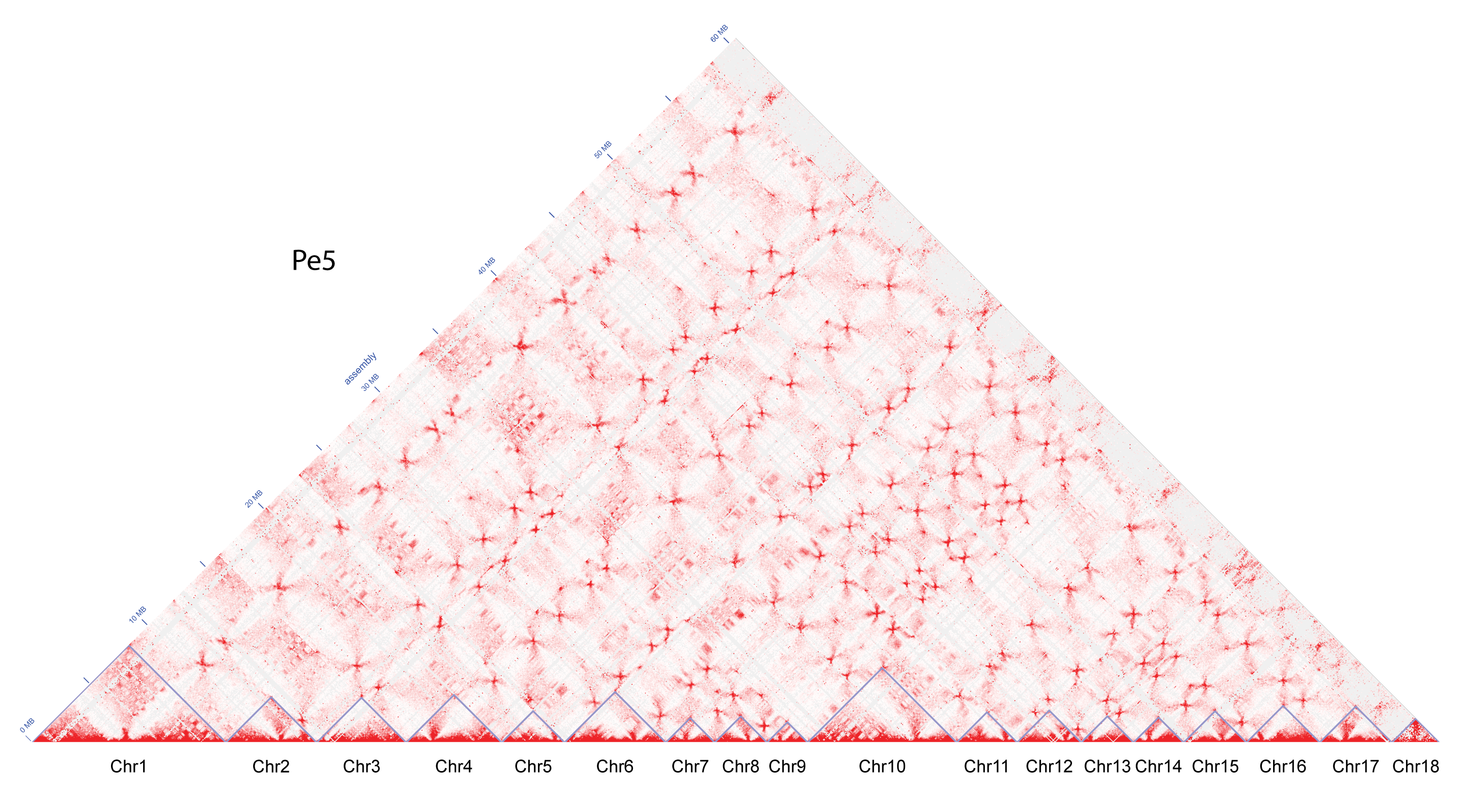

Supplement: S4 Fig — Hi-C heatmap displays the spatial interactions between chromosomes in the nucleus. Chromosome boundaries are indicated with blue lines. The observed interactions indicate a Rabl chromatin configuration with telomeric and centromeric regions inside the nucleus. (TIF) [file pgen.1011452.s004.tif]

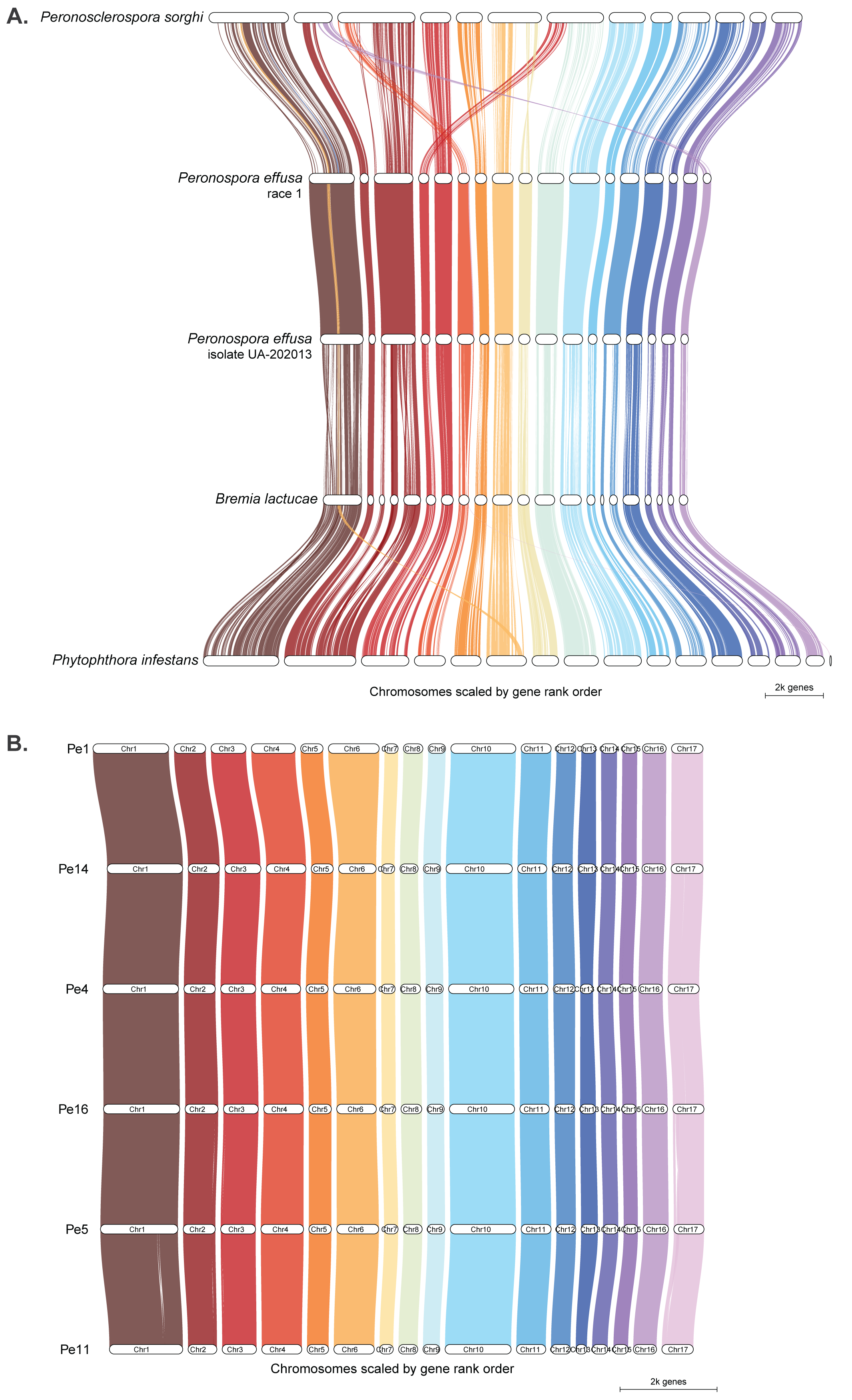

Supplement: S5 Fig — A. Comparison of six oomycete chromosome-level genome assemblies revealing conserved chromosome structure with a few chromosomal fusions. B. Comparison of our six chromosome-level genome assemblies for P. effusa revealing highly conserved chromosome structure with two rearrangements in chromosome in Pe11 and chromosome 17 in Pe5. (TIF) [file pgen.1011452.s005.tif]

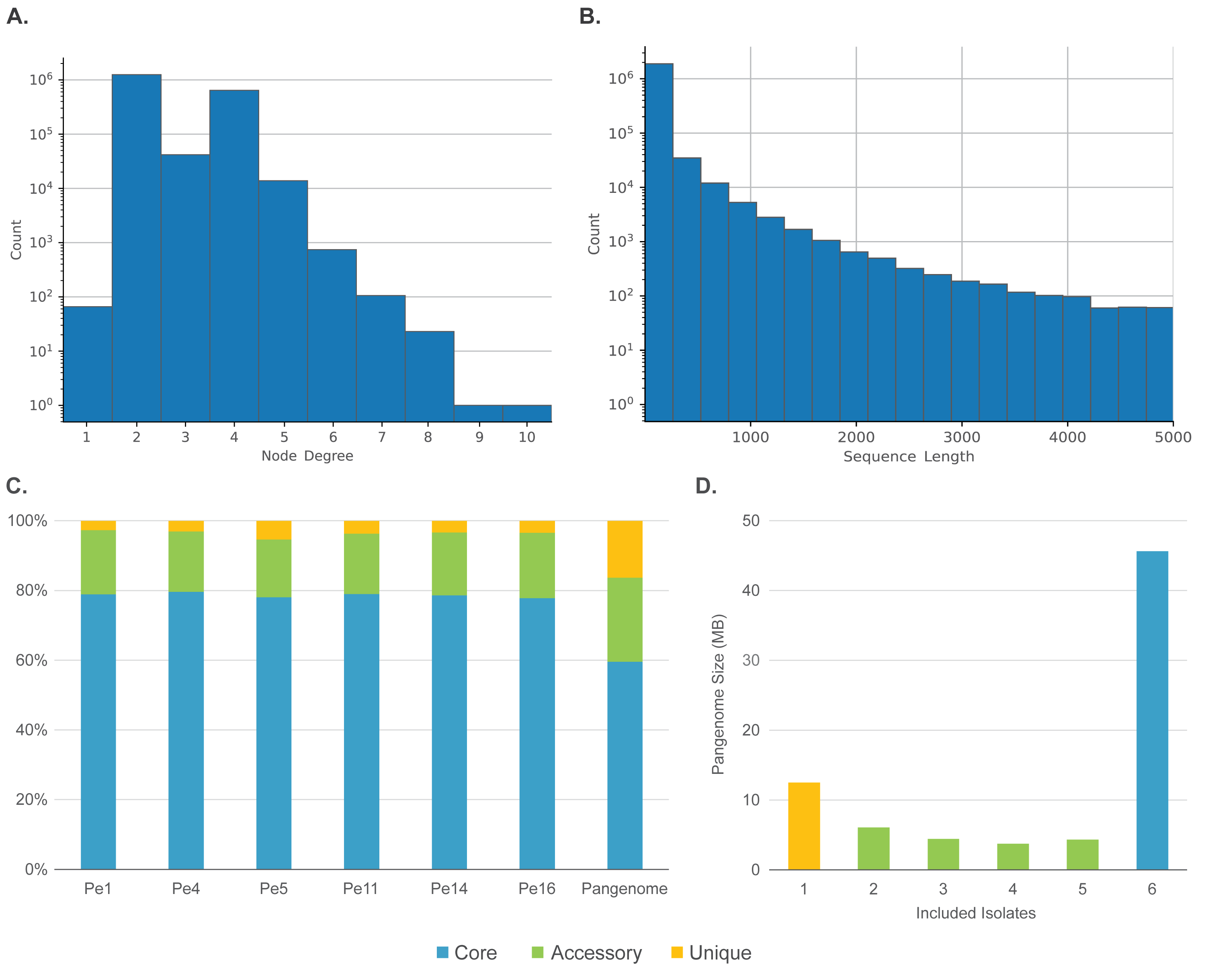

Supplement: S6 Fig — A. Histogram of the node degree, i.e. the number of connections, of each node of the pangenome graph. B. Histogram of the sequence length of each node of the pangenome graph up to a max length of 5 kb. C. Bar plot of the percentage of the genome size that is core, accessory, or unique for each isolate and the pangenome. D. Bar plot of the total size of pangenome graph nodes that belong from one to six isolates. (TIF) [file pgen.1011452.s006.tif]

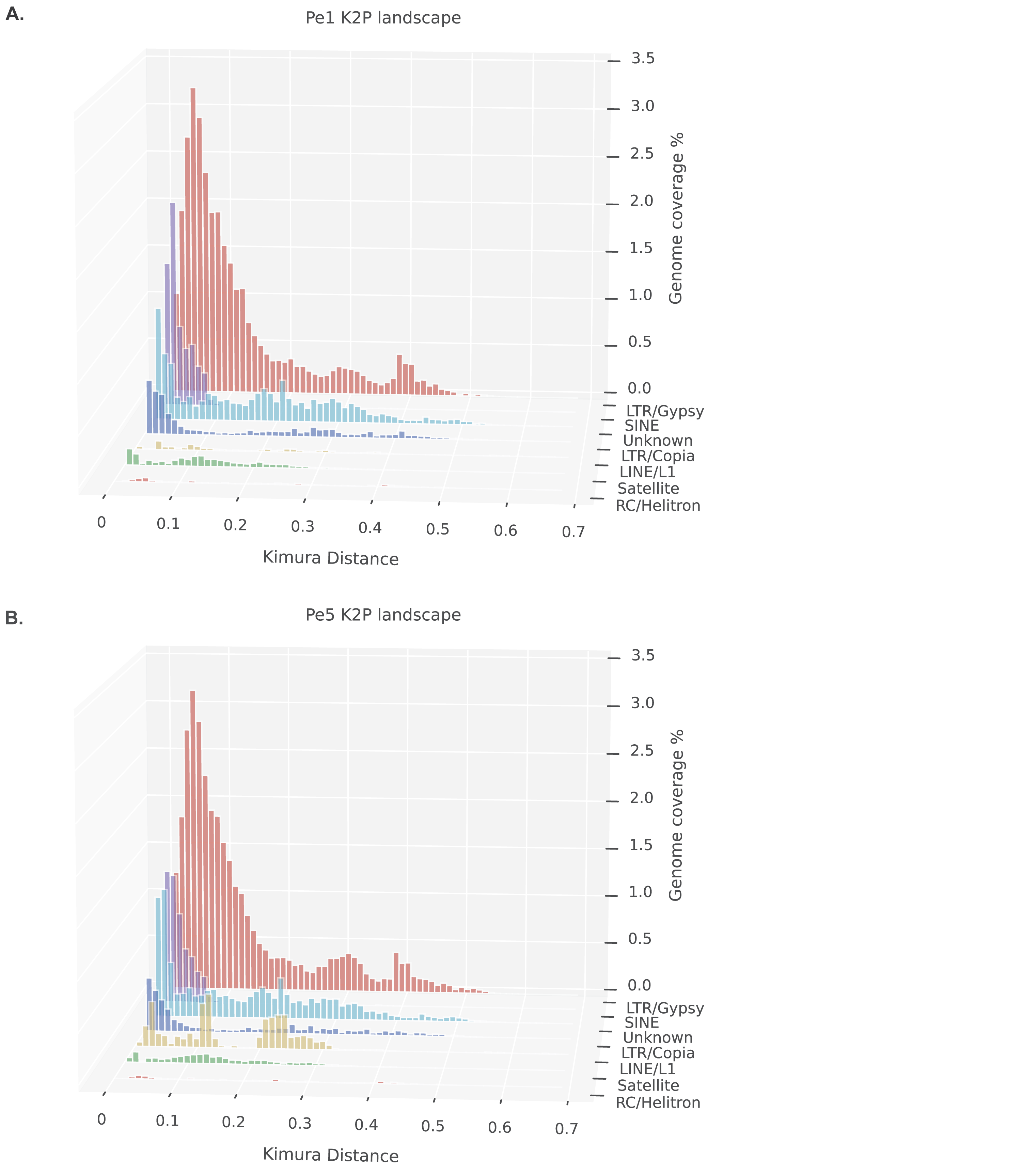

Supplement: S7 Fig — Kimura distance is the measure of divergence between individual TE copies and the corresponding TE consensus sequence (Kimura, 1980), i.e., the lower the Kimura distance, the more similar the copy is to the consensus and thus the more recent it was most likely copied. (TIF) [file pgen.1011452.s007.tif]

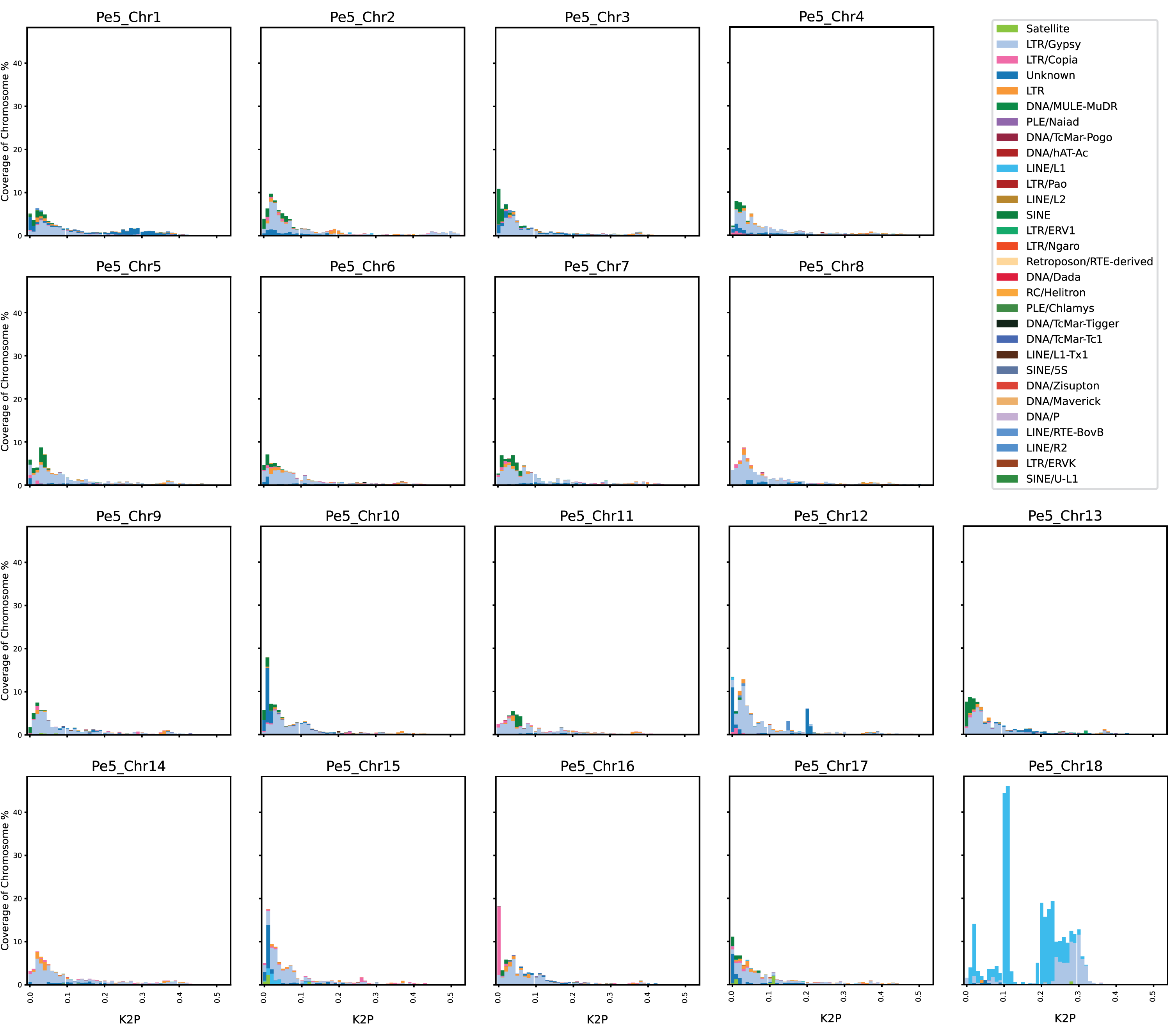

Supplement: S8 Fig — Kimura distance is the measure of divergence between individual TE copies and the corresponding TE consensus sequence (Kimura, 1980), i.e., the lower the Kimura distance, the more similar the copy is to the consensus and thus the more recent it was most likely copied. (TIF) [file pgen.1011452.s008.tif]

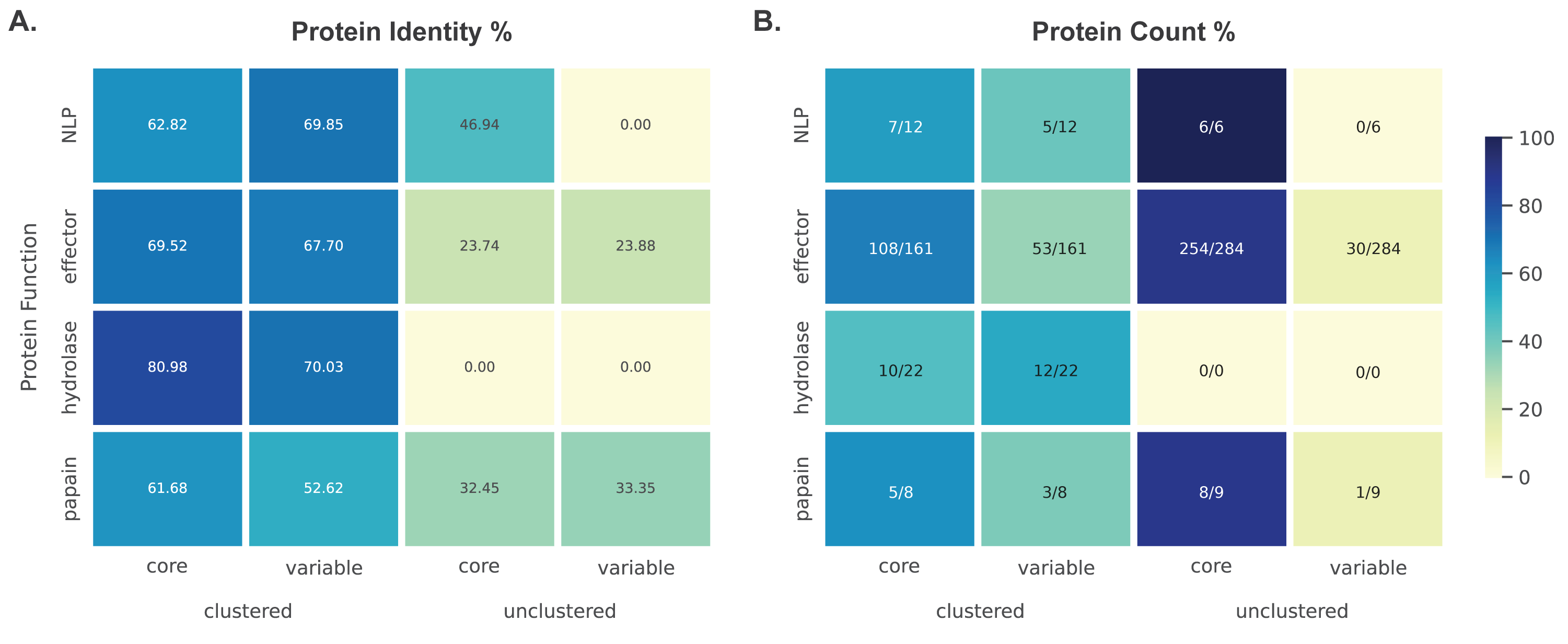

Supplement: S9 Fig — A. Heatmap of the average protein identity for each cluster and the average protein identity for all unclustered proteins. B. Heatmap of the percentage of proteins that are core or variable for each protein cluster and for all unclustered proteins. (TIF) [file pgen.1011452.s009.tif]

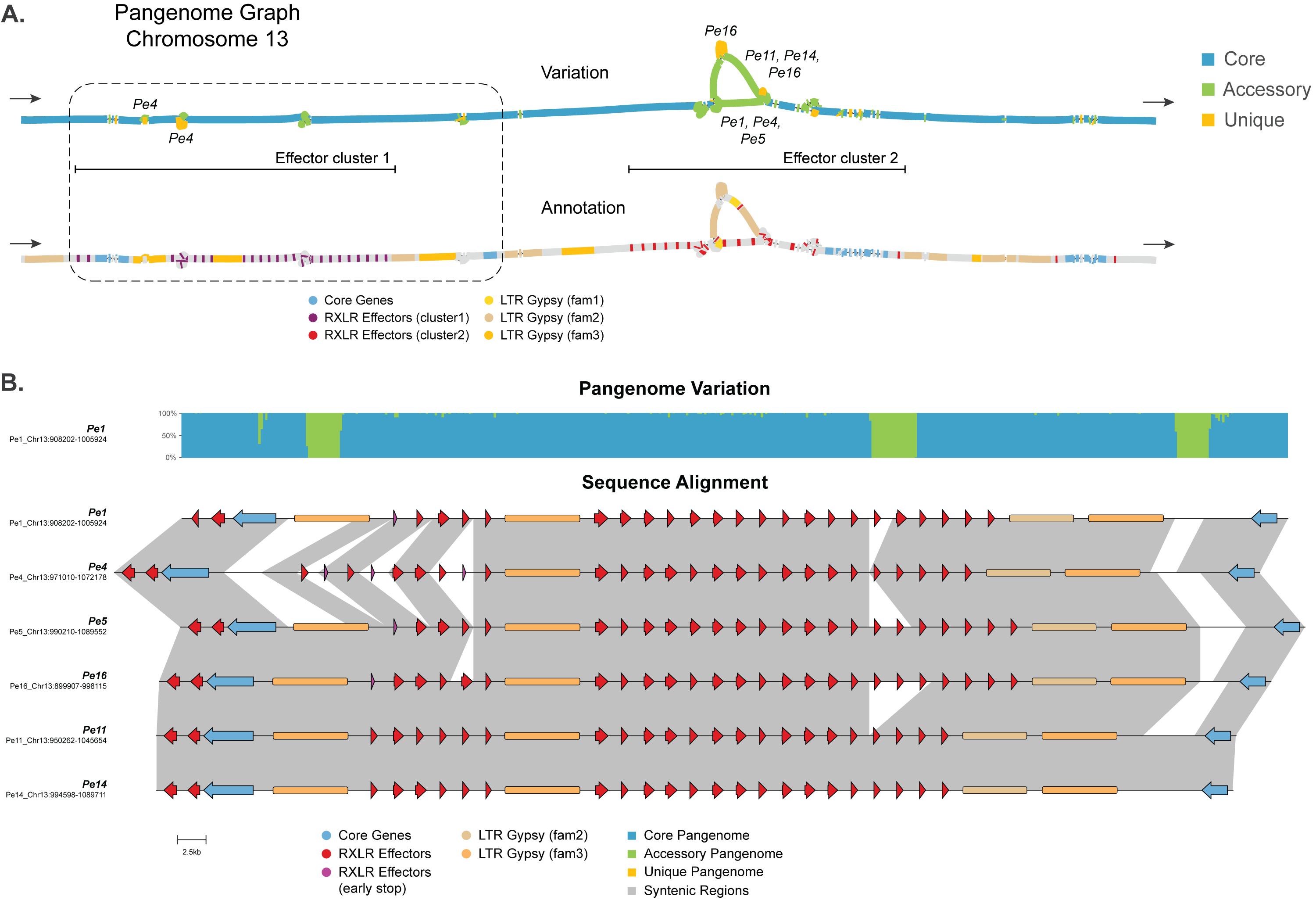

Supplement: S10 Fig — A. Part of chromosome 13, 0.2 Mb in size, is visualised by two pangenome graphs, with the start and end of the graph indicated with arrows. The first graph shows the variation between P. effusa isolates with core (blue), accessory (green), and unique (orange) regions. For large accessory and unique regions, the isolates that have these regions are indicated. The second graph visualises the effector genes belonging to the first (purple) and second (red) effector clusters on the graph. The highlighted area is expanded in B. Effector genes (red), pseudogenes (purple), and core genes (blue) are indicated as arrows, and LTR Gypsy repeats (fam3: orange, fam2: brown) are indicated as boxes. The pangenome variation for this region is visualised in a stacked bar plot (core blue, accessory green, and unique orange) and syntenic regions between P. effusa isolates are connected with grey ribbons. (TIF) [file pgen.1011452.s010.tif]

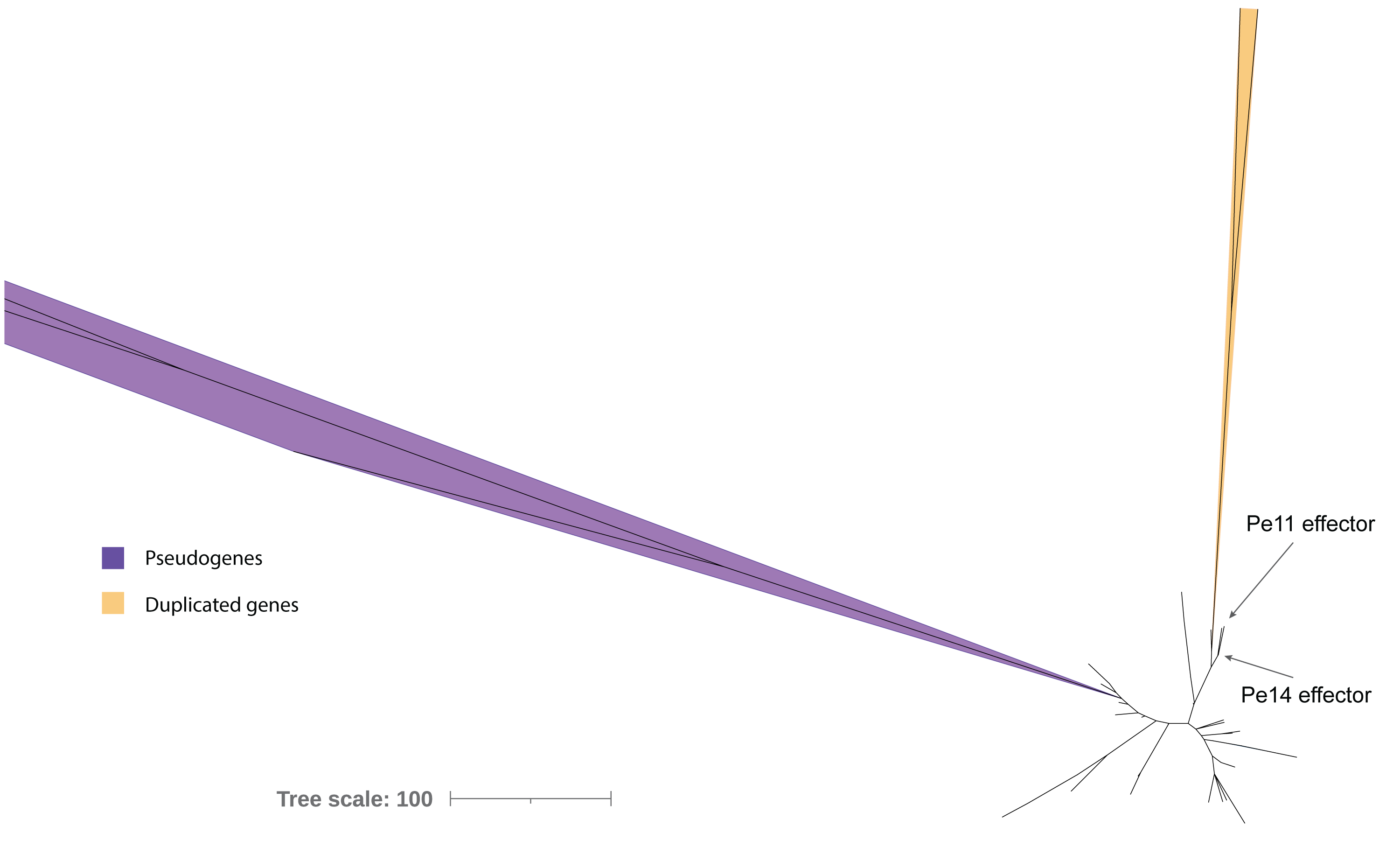

Supplement: S11 Fig — Nucleotide phylogeny of the 114 RXLR effector genes and five pseudogenes for the six P. effusa isolates in the second cluster of chromosome 13. The neighbour joining tree was built with IQ-TREE (v. 1.6.12) (Minh et al., 2020) and visualised with iTOL (v. 6.9) (Letunic & Bork, 2024). The location on the tree of the two genes downstream of the duplicated genes is indicated with arrows. (TIF) [file pgen.1011452.s011.tif]

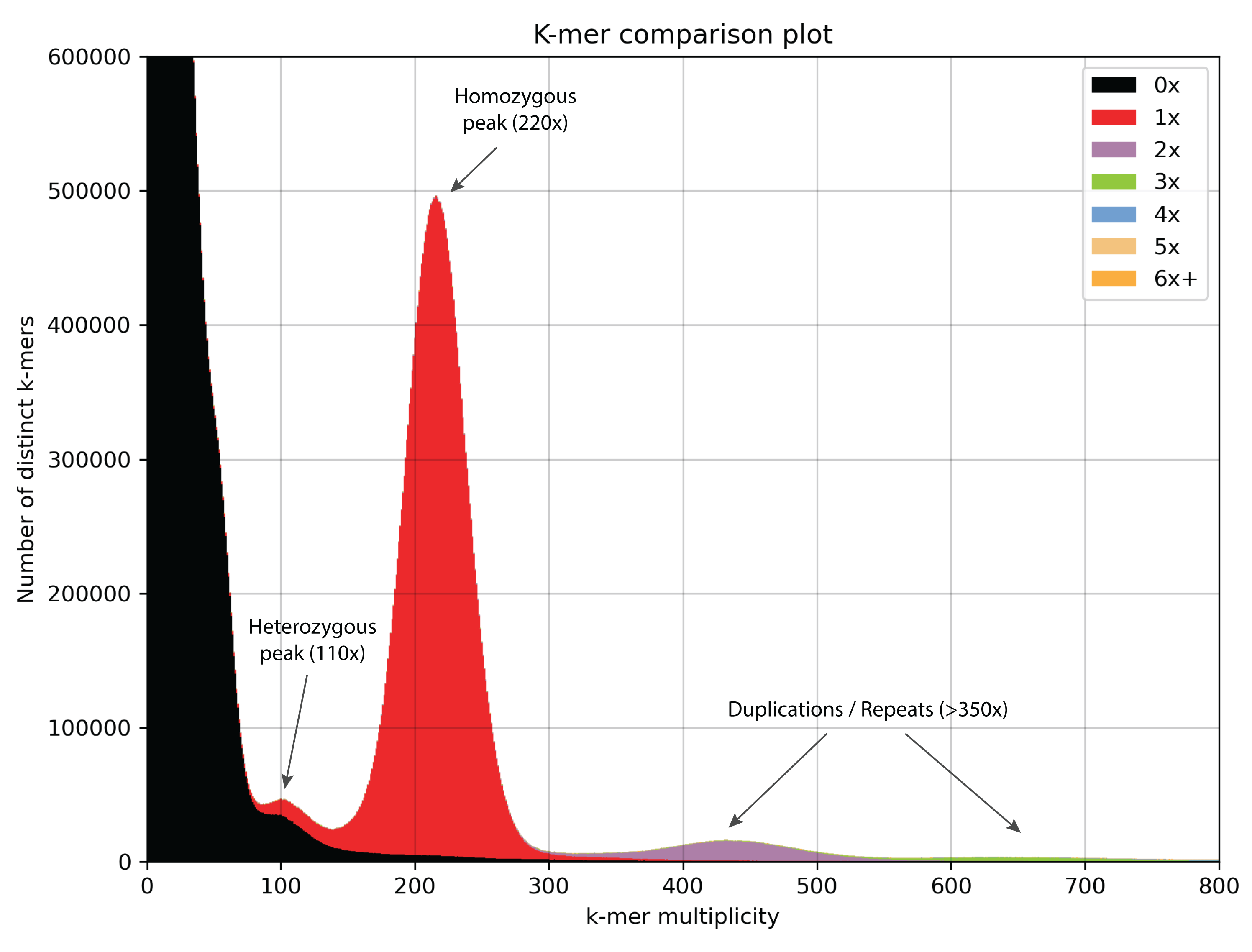

Supplement: S12 Fig — The short-read data of Pe1 were split into K-mers (size 27b) and their frequency is plotted using KAT (v. 2.4.2) (Mapleson et al., 2017). The K-mers are mapped to the genome assembly of Pe1 and the colour represents the number of copies that they are represented in the assembly. (TIF) [file pgen.1011452.s012.tif]

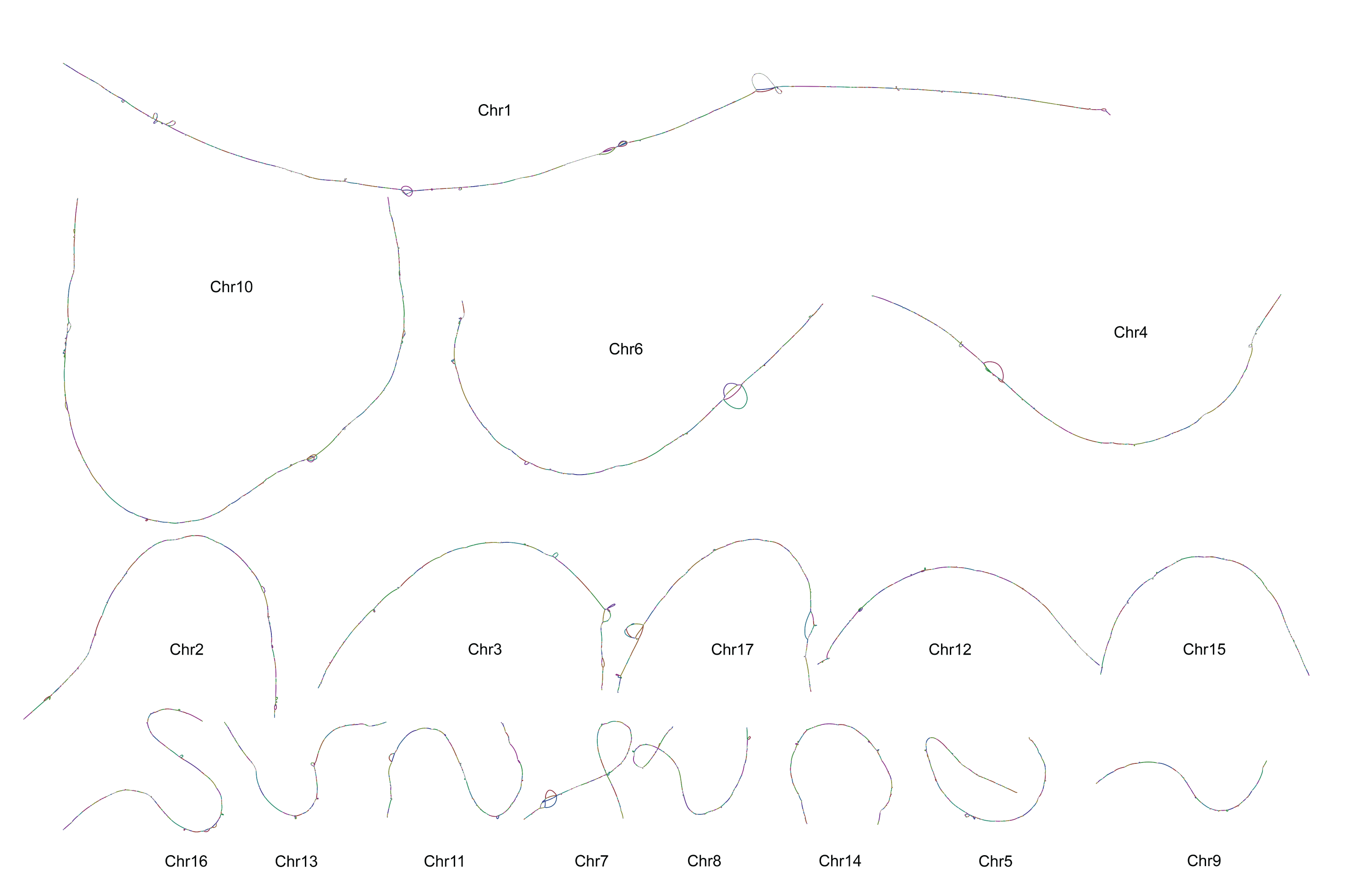

Supplement: S13 Fig — The pangenome graph was produced using the full genome assemblies of our six P. effusa isolates in a joint analysis, rather than generating separate pangenome graphs for each chromosome individually, which were then merged. Nodes are randomly coloured. (TIF) [file pgen.1011452.s013.tif]

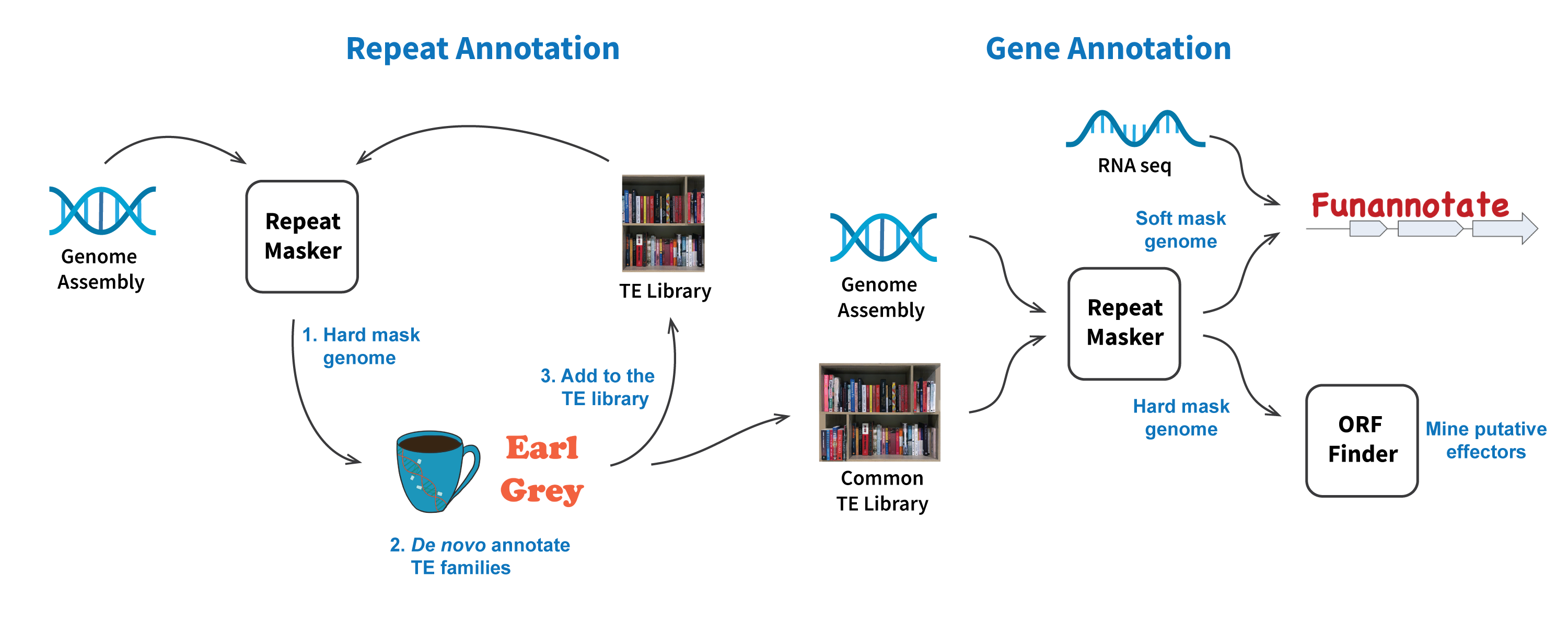

Supplement: S14 Fig — These structural annotations were then overlapped with the pangenome graph for reference-free genome annotation and whole-genome comparisons (Fig 2). (TIF) [file pgen.1011452.s014.tif]
