# Supplementary material for: Pangenome graph analysis reveals extensive effector copy-number variation in spinach downy mildew
Source: PLoS Genet. 2024 Oct 25;20(10):e1011452. doi: 10.1371/journal.pgen.1011452 (PMC11540230; doi:10.1371/journal.pgen.1011452)
Supplement: S1 Note — (DOCX) [file pgen.1011452.s018.docx]

## Note S1: Chromosome-level genome assemblies for six *Peronospora* *effusa* isolates

To obtain high-quality, chromosome-level genome assemblies, we produced on average, 12 Gb of Nanopore reads per *Peronospora effusa* isolate, with an N50 of 28.5 kb, and 8 Gb of Hi-C reads (S. Table 1). The Nanopore long-read data were assembled with Canu [1], and assembled contigs were subsequently filtered to remove the mitochondrial genome and bacterial contamination (Fig S2B), which is a known challenge for biotrophic plant pathogens [2,3], yielding 29-100 contigs per isolate. These contigs were further scaffolded into chromosomes using the chromatin contact information from Hi-C data (Fig S4), and remaining gaps were subsequently closed by manually joining contig overlaps that were supported by long reads. To correct for systematic sequencing errors of the Nanopore data, the chromosome-level genome assembly was polished with Illumina short-read data.

The quality and completeness of the assemblies were evaluated in a reference free method using short-read K-mer distribution and we observed that our haploid assemblies cover half of the heterozygous regions (1x) and cover fully the homozygous (2x), and highly repetitive regions (>3x) (Fig S12). We further evaluated the quality of the genome assemblies based on the coverage of Nanopore and Illumina reads along the assembled chromosomes, which support the chromosome structure with average coverage per isolate ranging from 53 - 271 reads (Fig S3); the number and constitution of the assembled chromosomes is also strongly supported by the Hi-C data (Fig S4). We did, however, also observed lower sequencing coverage for few long repetitive regions (longer than 100 kb), but the contiguity of these regions was supported by Nanopore reads that were longer than the respective region (Fig S3). The ribosomal RNA cluster at the beginning of chromosome 15 has five times higher Nanopore read coverage compared with the remainder of the genome, suggesting that this highly repetitive region is much longer than represented in the assembly.

## References

1. Koren S, Walenz BP, Berlin K, Miller JR, Bergman NH, Phillippy AM. Canu: Scalable and accurate long-read assembly via adaptive κ-mer weighting and repeat separation. Genome Res. 2017;27: 722–736. doi:10.1101/gr.215087.116

2. Strong MJ, Xu G, Morici L, Splinter Bon-Durant S, Baddoo M, Lin Z, et al. Microbial Contamination in Next Generation Sequencing: Implications for Sequence-Based Analysis of Clinical Samples. PLoS Pathog. 2014;10: e1004437. doi:10.1371/JOURNAL.PPAT.1004437

3. Klein J, Neilen M, van Verk M, Dutilh BE, van den Ackerveken G. Genome reconstruction of the non-culturable spinach downy mildew *Peronospora effusa* by metagenome filtering. PLoS ONE. 2020. doi:10.1371/journal.pone.0225808
