# Supplementary material for: Pangenome graph analysis reveals extensive effector copy-number variation in spinach downy mildew
Source: PLoS Genet. 2024 Oct 25;20(10):e1011452. doi: 10.1371/journal.pgen.1011452 (PMC11540230; doi:10.1371/journal.pgen.1011452)
Supplement: S2 Note — (DOCX) [file pgen.1011452.s019.docx]

## Note S2: Pangenome graph of six *Peronospora effusa* isolates enables comprehensive study of genome variation

The pangenome graph was generated separately for each chromosome, based on the Minigraph-Cactus pipeline, to aid the scalability of the pipeline [1]. We also created a pangenome graph using the whole genome assemblies of our six isolates as an input that resulted in a functionally identical graph with the 17 chromosomes fully separated (Fig S13). Using the whole genome rather than graphs for individual chromosomes is the appropriate approach for species that do not have conserved chromosome structure.

To annotate transposable elements (TEs), we applied a method for common TE discovery, where the library from the *de novo* TE discovery of *Pe1* was used to mask the genome of *Pe4*, before performing *de novo* TE discovery on *Pe4* and appending the common library with the new sequences discovered on *Pe4* (Fig S14). This method was recursively applied for *Pe5*, *Pe11*, *Pe14*, and *Pe16* resulting in a common TE library for all assembled isolates. This common TE library was subsequently used to annotate each genome assembly, which uncovered that between 50.58 and 52.4% of each genome is composed of repetitive sequences such as transposons, with long-terminal repeat (LTR) transposons being the most abundant superfamily covering around 40% of the genome (Fig 1C) (Materials and Methods – Transposable element annotation). We used repeat annotated genomes as a basis to structurally annotate genes with funannotate [2] by incorporating RNAseq data from *Pe1*, *Pe5*, *Pe11*, *Pe14*, *and Pe16* together with *ab initio* and homology-based gene annotation, which resulted in 9,869 to 10,008 protein-coding genes and in 6,092 to 7,362 tRNA genes.

To be able to consistently annotate genes for multiple *P. effusa* isolates and to overcome potential errors introduced by the separate gene annotation, we exploited the information in the pangenome graph to reannotate genome assemblies. To this end, we used the annotations of each isolate, the available RNAseq data, and the alignment of each genome to the pangenome graph to perform a common annotation using the Common-Annotation-Toolkit (Fig 2B, steps 1-3) (Materials and Methods – Pangenome graphs and common annotation) [1,3].

To compare the annotated protein-coding genes for each *P. effusa* isolate, we assigned all genes into groups based on their position on the pangenome graph, thus creating single-copy gene orthogroups based on synteny along the graph rather than the traditional approaches that group genes based on sequence similarity alone (Fig 2B, step 4). This method results in 12,379 orthogroups, of which 9,031 (73%) are conserved in the pangenome, while for each isolate 86.8 to 90.9% of the predicted protein-coding genes are conserved. Most unique genes are found in *Pe5* (4.3%, 450) and the lowest number in *Pe1* (1.1%, 110).

## References

1. Hickey G, Monlong J, Ebler J, Novak AM, Eizenga JM, Gao Y, et al. Pangenome graph construction from genome alignments with Minigraph-Cactus. Nature Biotechnology 2023. 2023; 1–11. doi:10.1038/S41587-023-01793-W

2. Palmer J. funannotate. 2017. Available: https://github.com/nextgenusfs/funannotate

3. Fiddes IT, Armstrong J, Diekhans M, Nachtweide S, Kronenberg ZN, Underwood JG, et al. Comparative Annotation Toolkit (CAT)—simultaneous clade and personal genome annotation. Genome Res. 2018;28: 1029–1038. doi:10.1101/GR.233460.117
